# Supplementary material for: The VelB IDD promotes selective heterodimer formation of velvet proteins for fungal development
Source: Life Sci Alliance. 2025 Nov 11;9(2):e202503395. doi: 10.26508/lsa.202503395 (PMC12614781; doi:10.26508/lsa.202503395)
Supplement: Supplementary file 3 [file LSA-2025-03395_TableS3.docx]

**Supplementary Tables**

**Table S3: Putative interaction partners identified in the GFP pull down of VelB-GFP and VelB^∆IDD^‑GFP expressing strains.**

| **Sys. name** | **Std. name** | **Description** | **VelB-GFP  LFQ** | **VelB^∆IDD^-GFP  LFQ** | **VelB-GFP**  **MS/MS** | **VelB^∆IDD^-GFP**  **MS/MS** | **VelB-GFP**  **Unique  peptides** | **VelB^∆IDD^-GFP**  **Unique  peptides** |
| --- | --- | --- | --- | --- | --- | --- | --- | --- |
| **AN0363** | **VelB** | **Bait protein** | 28.88 26.18 25.22 | 29.77 27.77 28.05 | 735 191 112 | 728 139 36 | 15 11 13 | 16 9 9 |
| **AN1052** | **VeA** | Protein involved in light-sensitive control of differentiation and secondary metabolism | 25.51 23.75 23.78 | 26.35 25.85 26.01 | 406 125 89 | 405 98 47 | 17 13 16 | 16 10 12 |
| **AN1959** | **VosA** | Nuclear protein involved in spore formation and trehalose accumulation | 24.32 22.03 16.89 | 14.92 15.86 15.24 | 166 8 1 | 1 0 0 | 11 3 1 | 1 0 0 |
| **AN9339** | **CatB** | Hyphal catalase with a predicted role in gluconic acid and gluconate metabolism | 23.9 20.69 23.05 | 22.7 22.65 23.39 | 168 8 30 | 125  40 46 | 15 4 15 | 16 10 17 |
|  | | | | | | | | |
| Translation | | | | | | | | |
| **AN0907** | **unchar.** | Putative 40S ribosomal protein S23 (Rps23), putative ortholog of *S. cerevisiae* RPS23A | 15.88 17.85 17.35 | 20.14 20.74 15.76 | 6 2 0 | 19 10 1 | 1 1 0 | 6 2 1 |
| **AN1345** | **unchar.** | Putative 40S ribosomal protein S22 (Rps22), putative ortholog of *S. cerevisiae* RPS22A | 16.39 17.58 16.24 | 19.39 20.05 15.41 | 1 0 0 | 15 10 2 | 1 0 0 | 2 2 1 |
| **AN0314** | **unchar.** | Putative aspartyl-tRNA synthetase, putative ortholog of *S. cerevisiae* DPS1 | 15.39 17.09 17.03 | 21.86 22.11 23.06 | 4 0 0 | 159 58 81 | 1 0 0 | 23 10 24 |
|  | | | | | | | | |
| Primary metabolism | | | | | | | | |
| **AN5226** | **AcpA** | Acetate permease A, involved in acetate uptake | 15.39 17.33 16.91 | 21.01 19.72 16.67 | 1 0 0 | 39 4 2 | 1 0 0 | 4 2 1 |
| **AN9180** | **unchar.** | Putative transketolase, putative ortholog of *A. fumigatus* TktA | 16.88 17.1 15.96 | 20.42 19.98 15.46 | 0 0 0 | 52 14 1 | 0 0 0 | 14 6 1 |
| **AN1967** | **PpoA** | Psi factor producing oxygenase A, responsible for the formation of the oxylipin psiBα | 15.06 17.36 17.84 | 20.98 18.32 20.6 | 0 0 0 | 86 5 17 | 0 0 0 | 23 3 11 |
|  | | | | | | | | |
| RNA maturation and processing | | | | | | | | |
| **AN7474** | **unchar.** | Has domain(s) with predicted RNA binding, putative ortholog of *S. cerevisiae* JSN1 | 16 17.14 16.1 | 20.65 20.73 21.04 | 0 0 0 | 51 13 15 | 0 0 0 | 16 7 9 |
|  | | | | | | | | |
| Membrane/cell wall | | | | | | | | |
| **AN5020** | **ArfB/**  **AdpA** | ADP ribosylation factor B, required for normal endocytosis and polarized growth | 15.05 17.41 16.55 | 21.33 21.80 19.1 | 0 0 0 | 49 19 4 | 0 0 0 | 4 3 3 |
| **AN9079** | **unchar.** | Putative ortholog of *N. crassa* Ham-10, with role in conidia formation | 15.24 16.91 16.94 | 20 20.13 21.26 | 0 0 0 | 50 50 36 | 0 0 0 | 18 7 20 |
| **AN2928** | **unchar.** | Putative cell wall protein, uncharacterized | 15.09 16.25 16.29 | 19.08 20.56 20.17 | 0 0 1 | 11 7 4 | 0 0 1 | 3 2  2 |
| **AN11139** | **AbpA** | Putative actin-binding protein A of the cortical actin patches | 14.88 16.39 17.13 | 19.15 19.68 20.67 | 3 0 2 | 46 21 13 | 1 0 1 | 11 5 6 |
| **AN3098** | **NsfA** | Putative secretory component, ortholog of *S. cerevisiae* SEC18, has similarity to mammalian N-ethylmaleimide-sensitive factor | 13.88 17.07 16.29 | 18.5 19.77 20.64 | 2 0 0 | 16 20 32 | 1 0 0 | 8 7 16 |
|  | | | | | | | | |
| Signaling | | | | | | | | |
| **AN3102** | **PhkA** | Putative histidine-containing phosphotransfer protein | 16.59 17.07 17.68 | 19.53 17.61 20.65 | 0 0 0 | 46 6 26 | 0 0 0 | 16 4 17 |
| **AN3422** | **Ste7** | MAP kinase kinase (MAPKK), component of a signaling module SteD-SteC-MkkB-MpkB that controls coordination of development and secondary metabolism | 14.02 17 17.42 | 20.75 20.75 20.57 | 1 0 0 | 69 20 15 | 1 0 0 | 11 5 7 |
| **AN2130** | **unchar.** | Putative Ras guanine-nucleotide exchange factor activity, putative ortholog of *S. cerevisiae* CDC25 | 16.26 17.22 17.95 | 19.97 20.83  21.07 | 0 0 0 | 51 26 16 | 0 0 0 | 10 8 9 |
| **AN7576** | **unchar.** | Predicted Rho GTPase activating protein, putative ortholog of *S. pombe* Rga1 | 14.98 16.97 16.95 | 19.23 20.01 19.66 | 0 0 0 | 32 18 14 | 0 0 0 | 8 7 9 |
| **AN8836** | **Cla4** | Predicted PAK (p21-activated kinase) family protein | 15.06 18.01 16.83 | 20.79 21.62 22.53 | 0 0 0 | 58 48 46 | 0 0 0 | 14 11 18 |
| **AN0463** | **unchar.** | Predicted Rac guanine nucleotide exchange factor, putative ortholog of *C. albicans* DCK2 | 14.68 16.46 17.32 | 18.9 20 21.76 | 0 0 0 | 42 18 24 | 0 0 0 | 9 9 23 |
|  | | | | | | | | |
| Cell compartments | | | | | | | | |
| **AN4207** | **unchar.** | Putative ortholog of *C. albicans* APL4*,* with role in endosomal transport, vesicle mediated transport and AP-1 adaptor complex | 14.12 17.72 16.76 | 19.94 20.71 21.41 | 0 0 0 | 44 26 26 | 0 0 0 | 11 9 12 |
| **AN0995** | **unchar.** | Putative CLASP family microtubule-associated protein, putative ortholog of *S. pombe* Peg1 | 14.86 17.08 17.33 | 18.97 19.8 20.4 | 0 0 0 | 18 15 14 | 0 0 0 | 9 4 11 |
| **AN3029** | **unchar.** | Putative AP-1 adaptor complex subunit beta, Putative ortholog of *C. albicans* APL2 | 15.7 17.65 17.42 | 20.13 20.67 21.54 | 1 0 0 | 38 36 30 | 1 0 0 | 10 9 16 |
| **AN0706** | **unchar.** | Ortholog(s) have role in ER to Golgi vesicle-mediated transport, putative ortholog of *C. albicans* USO1 | 15 17.79 16.9 | 20.22 20.38 22.36 | 0 0 1 | 61 17 42 | 0 0 1 | 19 6 24 |
| **AN4168** | **unchar.** | DUF500 and SH3 domain protein, putative ortholog of *S. pombe* actin cortical patch component Lsb4 | 16.7 17.45 17.45 | 19.95 15.67 20.16 | 0 0 0 | 37 0 14 | 0 0 0 | 7 0 7 |
|  | | | | | | | | |
| DNA binding | | | | | | | | |
| **AN0228** | **unchar.** | Putative ortholog of *S. cerevisiae* replication licensing factor MCM6 | 15.82 17.3 16.84 | 19.2 20.24 21.01 | 0 0 0 | 28 13 25 | 0 0 0 | 11 7 13 |
| **AN6070** | **unchar.** | Putative ortholog of *S. cerevisiae* replication licensing factor MCM4 | 14.52 17.26 16.69 | 18.67 19.74 20.55 | 0 0 0 | 17 13 14 | 0 0 0 | 8 6 10 |
| **AN2278** | **unchar.** | Putative ortholog of *N. crassa* SNF2-family ATP dependent chromatin remodeling factor Snf21 (Crf3-1) | 14.56 17.69 17.05 | 20.02 20.34 21.76 | 0 0 1 | 58 28 41 | 0 0 1 | 15 8 17 |
| **AN4187** | **unchar.** | Putative ortholog of *N. crassa* TBP associated factor (Cfr8-1) | 15.31 17.42 16.07 | 19.45 18.96 21.27 | 2 0 0 | 42 13 33 | 1 0 0 | 13 5 22 |
| **AN7222** | **unchar.** | NACHT domain containing protein | 15.09 16.34 15.09 | 22.49 21.55 23.74 | 0 0 0 | 181 47 84 | 0 0 0 | 42 16 35 |
| **AN5168** | **unchar.** | Putative NACHT and Ankyrin domain protein, putative ortholog of *S. pombe* Akr1 | 15.45 17.06 16.85 | 20.19 20.52 21.23 | 0 0 0 | 56 27 29 | 0 0 0 | 13 8 15 |
|  | | | | | | | | |
| Unknown function | | | | | | | | |
| **AN3005** | **unchar.** | Protein of unknown function | 15.17 16.81 17.11 | 19.2 20-9 20.87 | 0 0 0 | 27 26 36 | 0 0 0 | 11 8 16 |
| **AN11181** | **unchar.** | Protein of unknown function | 15.18 18.12 17.48 | 19.32 20.17 20.58 | 0 0 0 | 35 12 14 | 0 0 0 | 12 6 11 |
| **AN5423** | **unchar.** | Protein of unknown function | 13.8 17.05 18.02 | 21.07 16.94 20.47 | 0 0 0 | 68 0 6 | 0 0 0 | 13 0 4 |

Proteins were identified in at least two out of three biological repetitions with MS/MS counts ≥ 4, unique peptides ≥ 3 and LFQ intensity ≥ 20 and which were absent in the control strain. Proteins with underlined AN numbers contain a nuclear localization signal predicted with cNLS mapper [2] with a score ≤ 5 (indicating a localization in both, nucleus and cytoplasm), Sys. Name = systematic name, Std. name = standard name, unchar. = uncharacterized. Descriptions were obtained and adapted from FungiDB and NCBI [3,4].

**References**

1. Ward JJ, McGuffin LJ, Bryson K, Buxton BF, Jones DT. The DISOPRED server for the prediction of protein disorder. *Bioinformatics*. 2004;20: 2138–2139. doi:10.1093/bioinformatics/bth195

2. Kosugi S, Hasebe M, Tomita M, Yanagawa H. Systematic identification of cell cycle-dependent yeast nucleocytoplasmic shuttling proteins by prediction of composite motifs. *Proceedings of the National Academy of Sciences*. 2009;106: 10171–10176. doi:10.1073/pnas.0900604106

3. Alvarez-Jarreta J, Amos B, Aurrecoechea C, Bah S, Barba M, Barreto A, et al. VEuPathDB: the eukaryotic pathogen, vector and host bioinformatics resource center in 2023. *Nucleic Acids Res*. 2024;52: D808–D816. doi:10.1093/NAR/GKAD1003

4. Sayers EW, Bolton EE, Brister JR, Canese K, Chan J, Comeau DC, et al. Database resources of the national center for biotechnology information. *Nucleic Acids Res*. 2022;50: D20–D26. doi:10.1093/NAR/GKAB1112

5. Harrison PW, Amode MR, Austine-Orimoloye O, Azov AG, Barba M, Barnes I, et al. Ensembl 2024. Nucleic Acids Res. 2024;52: D891–D899. doi:10.1093/nar/gkad1049

6. Sanchez JF, Entwistle R, Corcoran D, Oakley BR, Wang CCC. Identification and molecular genetic analysis of the cichorine gene cluster in *Aspergillus nidulans*. *Medchemcomm*. 2012;3: 997–1002. doi:10.1039/C2MD20055D

7. Bok JW, Chiang Y-M, Szewczyk E, Reyes-Dominguez Y, Davidson AD, Sanchez JF, et al. Chromatin-level regulation of biosynthetic gene clusters. *Nat Chem Biol*. 2009;5: 462–464. doi:10.1038/nchembio.177

8. Lo HC, Entwistle R, Guo CJ, Ahuja M, Szewczyk E, Hung JH, et al. Two separate gene clusters encode the biosynthetic pathway for the meroterpenoids austinol and dehydroaustinol in *Aspergillus nidulans*. *J Am Chem Soc.* 2012;134: 4709–4720. doi:10.1021/ja209809t

9. Nielsen ML, Nielsen JB, Rank C, Klejnstrup ML, Holm DK, Brogaard KH, et al. A genome-wide polyketide synthase deletion library uncovers novel genetic links to polyketides and meroterpenoids in *Aspergillus nidulans*. *FEMS Microbiol Lett.* 2011;321: 157–166. doi:https://doi.org/10.1111/j.1574-6968.2011.02327.x

10. Chiang Y-M, Szewczyk E, Nayak T, Davidson AD, Sanchez JF, Lo H-C, et al. Molecular Genetic Mining of the *Aspergillus* Secondary Metabolome: Discovery of the Emericellamide Biosynthetic Pathway. Chem Biol. 2008;15: 527–532. doi:https://doi.org/10.1016/j.chembiol.2008.05.010

11. Yu JH, Leonard TJ. Sterigmatocystin biosynthesis in *Aspergillus nidulans* requires a novel type I polyketide synthase. *J Bacteriol*. 1995;177: 4792–4800. doi:10.1128/JB.177.16.4792-4800.1995

12. Bouhired S, Weber M, Kempf-Sontag A, Keller NP, Hoffmeister D. Accurate prediction of the *Aspergillus nidulans* terrequinone gene cluster boundaries using the transcriptional regulator LaeA. *Fungal Genetics and Biology*. 2007;44: 1134–1145. doi:https://doi.org/10.1016/j.fgb.2006.12.010

13. Sanchez JF, Entwistle R, Hung J-H, Yaegashi J, Jain S, Chiang Y-M, et al. Genome-based deletion analysis reveals the prenyl xanthone biosynthesis pathway in *Aspergillus nidulans*. *J Am Chem Soc*. 2011;133: 4010–7. doi:10.1021/ja1096682

14. Ahmed AM, Ibrahim AM, Yahia R, Shady NH, Mahmoud BK, Abdelmohsen UR, et al. Evaluation of the anti-infective potential of the seed endophytic fungi of Corchorus olitorius through metabolomics and molecular docking approach. *BMC Microbiol*. 2023;23: 1–19. doi:10.1186/S12866-023-03092-5/FIGURES/11

15. Perlatti B, Lan N, Jiang Y, An Z, Bills G. Identification of Secondary Metabolites from *Aspergillus pachycristatus* by Untargeted UPLC-ESI-HRMS/MS and Genome Mining. Molecules. 2020;25. doi:10.3390/MOLECULES25040913

16. Liu L, Sasse C, Dirnberger B, Valerius O, Fekete-Szücs E, Harting R, et al. Secondary metabolites of hülle cells mediate protection of fungal reproductive and overwintering structures against fungivorous animals. *Elife*. 2021;10. doi:10.7554/ELIFE.68058

17. Thieme KG, Gerke J, Sasse C, Valerius O, Thieme S, Karimi R, et al. Velvet domain protein VosA represses the zinc cluster transcription factor SclB regulatory network for *Aspergillus nidulans* asexual development, oxidative stress response and secondary metabolism. *PLoS Genet*. 2018;14: e1007511. doi:10.1371/journal.pgen.1007511

18. Kralj A, Kehraus S, Krick A, Eguereva E, Kelter G, Maurer M, et al. Arugosins G and H: prenylated polyketides from the marine-derived fungus *Emericella nidulans* var. acristata. *J Nat Prod*. 2006;69: 995–1000. doi:10.1021/NP050454F

19. Nielsen KF, Månsson M, Rank C, Frisvad JC, Larsen TO. Dereplication of microbial natural products by LC-DAD-TOFMS. *J Nat Prod*. 2011;74: 2338–2348. doi:10.1021/NP200254T/SUPPL_FILE/NP200254T_SI_001.ZIP

20. Hamed AA, El-Shiekh RA, Mohamed OG, Aboutabl EA, Fathy FI, Fawzy GA, et al. Cholinesterase Inhibitors from an Endophytic Fungus *Aspergillus niveus* Fv-er401: Metabolomics, Isolation and Molecular Docking. *Molecules*. 2023;28: 2559. doi:10.3390/MOLECULES28062559/S1

21. Chiang YM, Szewczyk E, Nayak T, Davidson AD, Sanchez JF, Lo HC, et al. Molecular genetic mining of the *Aspergillus* secondary metabolome: discovery of the emericellamide biosynthetic pathway. *Chem Biol.* 2008;15: 527–532. doi:10.1016/J.CHEMBIOL.2008.05.010

22. McCluskey K, Wiest A, Plamann M. The Fungal Genetics Stock Center: a repository for 50 years of fungal genetics research. J Biosci. 2010;35: 119–26.

23. Bayram Ö, Bayram ÖS, Ahmed YL, Maruyama J, Valerius O, Rizzoli SO, et al. The *Aspergillus nidulans* MAPK module AnSte11-Ste50-Ste7-Fus3 controls development and secondary metabolism. *PLoS Genet.* 2012;8: e1002816. doi:10.1371/journal.pgen.1002816

24. Fradin EF, Zhang Z, Juarez Ayala JC, Castroverde CDM, Nazar RN, Robb J, et al. Genetic dissection of *Verticillium* wilt resistance mediated by tomato Ve1*. Plant Physiol*. 2009;150: 320–32. doi:10.1104/pp.109.136762

25. Höfer AM, Harting R, Aßmann NF, Gerke J, Schmitt K, Starke J, et al. The velvet protein Vel1 controls initial plant root colonization and conidia formation for xylem distribution in *Verticillium* wilt. *PLoS Genet*. 2021;17. doi:10.1371/JOURNAL.PGEN.1009434

26. Ahmed YL, Gerke J, Park H-S, Bayram Ö, Neumann P, Ni M, et al. The Velvet family of fungal regulators contains a DNA-binding domain structurally similar to NF-κB. *PLoS Biol*. 2013;11: e1001750. doi:10.1371/journal.pbio.1001750

27. Jöhnk B, Bayram Ö, Abelmann A, Heinekamp T, Mattern DJ, Brakhage AA, et al. SCF ubiquitin ligase F-box protein Fbx15 controls nuclear co-repressor localization, stress response and virulence of the human pathogen *Aspergillus fumigatus*. *PLoS Pathog*. 2016;12: e1005899. doi:10.1371/journal.ppat.1005899

28. Gerke J, Köhler AM, Wennrich J-P, Große V, Shao L, Heinrich AK, et al. Biosynthesis of Antibacterial Iron-Chelating Tropolones in *Aspergillus nidulans* as Response to Glycopeptide-Producing Streptomycetes. *Frontiers in Fungal Biology*. 2022;2. doi:10.3389/ffunb.2021.777474

29. Leonard M, Kühn A, Harting R, Maurus I, Nagel A, Starke J, et al. *V. longisporum* elicits media-dependent secretome responses with a further capacity to distinguish between plant-related environments. *bioRxiv*. 2020; 2020.02.11.943803. doi:10.1101/2020.02.11.943803

30. Park H-S, Nam T-Y, Han K-H, Kim SC, Yu J-H. VelC Positively Controls Sexual Development in *Aspergillus nidulans. PLoS One*. 2014;9: e89883. doi:10.1371/journal.pone.0089883

31. Covert SF, Kapoor P, Lee M, Briley A, Nairn CJ. *Agrobacterium tumefaciens*-mediated transformation of *Fusarium circinatum*. Mycol Res. 2001;105: 259–264. doi:10.1017/S0953756201003872
