# Supplementary material for: The VelB IDD promotes selective heterodimer formation of velvet proteins for fungal development
Source: Life Sci Alliance. 2025 Nov 11;9(2):e202503395. doi: 10.26508/lsa.202503395 (PMC12614781; doi:10.26508/lsa.202503395)
Supplement: Supplementary file 9 [file LSA-2025-03395_TableS9.docx]

**Supplementary Tables**

**Table S9: Oligonucleotides used for amplifications and plasmid constructions.**

| **Name** | **5’ – sequence – 3’** | **Gene amplification** |
| --- | --- | --- |
| AO74 | TGC TTG CCA TCT TGC TAC ACC | 5’ + *vel2^ΔIDD^:GFP* |
| AO75 | GTA CAT CCC CTG AGG GTG GCG GAC GAG GTT CAC CT | 5’ + *vel2^ΔIDD^:GFP* |
| AO76 | CCT CAG GGG ATG TAC ACG | *vel2^ΔIDD^:GFP* |
| AO77 | CTA ATA ATC GTC ATC GTC GT | *vel2^ΔIDD^:GFP* |
| AO78 | TTT TCT AGA ATG AGG CGC TGG TCA TTC AA | 3’ *vel2^ΔIDD^:GFP* |
| AO79 | TAG TCT AGA TGC ATC TTC AGA CAC GCA AA | 3’ *vel2^ΔIDD^:GFP* |
| AO165 | GGT GGT AGC GGT GGT GT | *GFP* |
| AO167 | ATT CTT AAT TAA GAT TGC TTG CCA TCT TGC TAC ACC | 5’ *vel2^ΔIDD^:GFP* |
| AO168 | ACC ACC GCT ACC ACC ATA ATC GTC ATC GTC GTC A | 5’ + *vel2^ΔIDD^:GFP* |
| AO169 | AGG TAA TCC TTC TTT CTA GAA TGA GGC GCT GGT | 3’ *vel2^ΔIDD^:GFP* |
| AO170 | AGG ACT TCT AGA AGG TGC ATC TTC AGA CAC GCA | 3’ *vel2^ΔIDD^:GFP* |
| JG45 | CCC CAT GGC GAT GTA CGC TGT TGA GGA TAG G | *velB^∆IDD^*:*his* |
| JG46 | CCC TCG AGG TAT TCG TTA TCC AGA CCA TC | *velB^∆IDD^*:*his* |
| KT142 | ATA ATA TGG CCA TCT AAG AAT TCT GCC GGC GTT TAT TTG | 3’ *veA:ha* |
| KT163 | CTA TAG GCC TGA GTG TTA GGC GTA GTC GGG GAC G | *ha* |
| KT166 | CAA AGC CTG CCA CCA TGC GTT ACC CCT ACG ACG TCC CCG ACT ACG CCT AA | 5’ + veA:ha |
| KT197 | ATC GAT AAG CTT GAT GTT TAA ACT GGA GTG CCT TTC GTC | 5’ + *veA:ha* |
| KT198 | CTG CAG GAA TTC GAT GTT TAA ACA TTC TGG CTC GTC TGC | 3’ *veA:ha* |
| RH514 | ACC GGT CAC TGT ACA TTA CTT GTA CAG CTC GTC CAT | *GFP* |
| RH590 | TGT ACA GTG ACC GGT GA | hygromycin resistance cassette |
| RO4 | AAA GAA GGA TTA CCT CTA AAC AA | hygromycin resistance cassette |
| SR05 | CTG CAG GAA TTC GAT GTT TAA ACC GTG CAG TCA GTC TAC CTA C | 5’ + *velB* |
| SR07 | ATA ATA TGG CCA TCT AGA CCG TAT ATT GTT TCA TAA ATC C | 3’ *velB* |
| SR08 | ATC GAT AAG CTT GAT GTT TAA ACC CGC TGT ACA TGT AAT GTC CG | 3’ *velB* |
| SR18 | GGT GGT AGC GGT GGT GTG AGC AAG GGC GAG GAG | *gfp* |
| SR20 | CTA TAG GCC TGA GTG CTA CTT GTA CAGT TCG TCC ATG C | *gfp* |
| SR24 | ACC ACC GCT ACC ACC GTA TTC GTT ATC CAG ACC ATC G | 5’ + *velB* |
| SR49 | ATA ATA TGG CCA TCT GGA TTC TCG TTT GTG GAA CAC | 5’ *vosA:ha* |
| SR75 | ATC GAT AAG CTT GAT GTT TAA ACT TTC CGT AGG TCG ATC C | 5’ *vosA:ha* |
| SR76 | AGG AAT TCG ATG TTT AAA CAA GGG CTC CTG TCG GA | 5’ + *vosA:ha* |
| SR108 | GGC ATG TTC ACG CGC AAT CT | *velB^∆IDD^* |
| SR109 | AGA TTG CGC GTG AAC ATG CCG TGC TTC ACA AGA TTT ACT TCA TG | 5’ + *velB^∆IDD^* |
| SR110 | AGG AAT TCG ATG TTT AAA CGA TTA GGA GAA GTC CAC TTT | 5’ + *velB^∆IDD^* |
| SR111 | TTG ACC TAT AGG CCT TTA GTA TTC GTT ATC CAG ACC ATC | *velB^∆IDD^* |
| SR112 | ATA ATA TGG CCA TCT AGA CCG TAT ATT GTT TCA TAA ATC C | 3’ *velB^∆IDD^* |
| SR113 | ATA AGC TTG ATG TTT AAA CCG CTC GCG CCC CAT | 3’ *velB^∆IDD^* |
| SR201 | CTA TAG GCC TGA GTG TTA GGC GTA GTC GGG GAC GTC GTA GGG GTA CCG AGG AGT TCC GTT CGC | 5’ + *vosA:ha* |
| SR253 | GTG CTT CAC AAG ATT TAC TTC ATG | 5’ + *velB^AfIDD^* |
| SR254 | AAG TAA ATC TTG TGA AGC ACC AGC GCG AGG TGA ACC TC | IDD of *Vdvel2* |
| SR255 | AGA TTG CGC GTG AAC ATG CCC TGA GGC GCA CCT GCG | IDD of *Vdvel2* |
| SR266 | AAG TAA ATC TTG TGA AGC ACT CAG CCA CGT CGC CAT CAA TAT C | IDD of *AfvelB* |
| SR267 | AGA TTG CGC GTG AAC ATG CCA CCG GGC CCC GCC GGA A | IDD of *AfvelB* |

**References**

1. Ward JJ, McGuffin LJ, Bryson K, Buxton BF, Jones DT. The DISOPRED server for the prediction of protein disorder. *Bioinformatics*. 2004;20: 2138–2139. doi:10.1093/bioinformatics/bth195

2. Kosugi S, Hasebe M, Tomita M, Yanagawa H. Systematic identification of cell cycle-dependent yeast nucleocytoplasmic shuttling proteins by prediction of composite motifs. *Proceedings of the National Academy of Sciences*. 2009;106: 10171–10176. doi:10.1073/pnas.0900604106

3. Alvarez-Jarreta J, Amos B, Aurrecoechea C, Bah S, Barba M, Barreto A, et al. VEuPathDB: the eukaryotic pathogen, vector and host bioinformatics resource center in 2023. *Nucleic Acids Res*. 2024;52: D808–D816. doi:10.1093/NAR/GKAD1003

4. Sayers EW, Bolton EE, Brister JR, Canese K, Chan J, Comeau DC, et al. Database resources of the national center for biotechnology information. *Nucleic Acids Res*. 2022;50: D20–D26. doi:10.1093/NAR/GKAB1112

5. Harrison PW, Amode MR, Austine-Orimoloye O, Azov AG, Barba M, Barnes I, et al. Ensembl 2024. Nucleic Acids Res. 2024;52: D891–D899. doi:10.1093/nar/gkad1049

6. Sanchez JF, Entwistle R, Corcoran D, Oakley BR, Wang CCC. Identification and molecular genetic analysis of the cichorine gene cluster in *Aspergillus nidulans*. *Medchemcomm*. 2012;3: 997–1002. doi:10.1039/C2MD20055D

7. Bok JW, Chiang Y-M, Szewczyk E, Reyes-Dominguez Y, Davidson AD, Sanchez JF, et al. Chromatin-level regulation of biosynthetic gene clusters. *Nat Chem Biol*. 2009;5: 462–464. doi:10.1038/nchembio.177

8. Lo HC, Entwistle R, Guo CJ, Ahuja M, Szewczyk E, Hung JH, et al. Two separate gene clusters encode the biosynthetic pathway for the meroterpenoids austinol and dehydroaustinol in *Aspergillus nidulans*. *J Am Chem Soc.* 2012;134: 4709–4720. doi:10.1021/ja209809t

9. Nielsen ML, Nielsen JB, Rank C, Klejnstrup ML, Holm DK, Brogaard KH, et al. A genome-wide polyketide synthase deletion library uncovers novel genetic links to polyketides and meroterpenoids in *Aspergillus nidulans*. *FEMS Microbiol Lett.* 2011;321: 157–166. doi:https://doi.org/10.1111/j.1574-6968.2011.02327.x

10. Chiang Y-M, Szewczyk E, Nayak T, Davidson AD, Sanchez JF, Lo H-C, et al. Molecular Genetic Mining of the *Aspergillus* Secondary Metabolome: Discovery of the Emericellamide Biosynthetic Pathway. Chem Biol. 2008;15: 527–532. doi:https://doi.org/10.1016/j.chembiol.2008.05.010

11. Yu JH, Leonard TJ. Sterigmatocystin biosynthesis in *Aspergillus nidulans* requires a novel type I polyketide synthase. *J Bacteriol*. 1995;177: 4792–4800. doi:10.1128/JB.177.16.4792-4800.1995

12. Bouhired S, Weber M, Kempf-Sontag A, Keller NP, Hoffmeister D. Accurate prediction of the *Aspergillus nidulans* terrequinone gene cluster boundaries using the transcriptional regulator LaeA. *Fungal Genetics and Biology*. 2007;44: 1134–1145. doi:https://doi.org/10.1016/j.fgb.2006.12.010

13. Sanchez JF, Entwistle R, Hung J-H, Yaegashi J, Jain S, Chiang Y-M, et al. Genome-based deletion analysis reveals the prenyl xanthone biosynthesis pathway in *Aspergillus nidulans*. *J Am Chem Soc*. 2011;133: 4010–7. doi:10.1021/ja1096682

14. Ahmed AM, Ibrahim AM, Yahia R, Shady NH, Mahmoud BK, Abdelmohsen UR, et al. Evaluation of the anti-infective potential of the seed endophytic fungi of Corchorus olitorius through metabolomics and molecular docking approach. *BMC Microbiol*. 2023;23: 1–19. doi:10.1186/S12866-023-03092-5/FIGURES/11

15. Perlatti B, Lan N, Jiang Y, An Z, Bills G. Identification of Secondary Metabolites from *Aspergillus pachycristatus* by Untargeted UPLC-ESI-HRMS/MS and Genome Mining. Molecules. 2020;25. doi:10.3390/MOLECULES25040913

16. Liu L, Sasse C, Dirnberger B, Valerius O, Fekete-Szücs E, Harting R, et al. Secondary metabolites of hülle cells mediate protection of fungal reproductive and overwintering structures against fungivorous animals. *Elife*. 2021;10. doi:10.7554/ELIFE.68058

17. Thieme KG, Gerke J, Sasse C, Valerius O, Thieme S, Karimi R, et al. Velvet domain protein VosA represses the zinc cluster transcription factor SclB regulatory network for *Aspergillus nidulans* asexual development, oxidative stress response and secondary metabolism. *PLoS Genet*. 2018;14: e1007511. doi:10.1371/journal.pgen.1007511

18. Kralj A, Kehraus S, Krick A, Eguereva E, Kelter G, Maurer M, et al. Arugosins G and H: prenylated polyketides from the marine-derived fungus *Emericella nidulans* var. acristata. *J Nat Prod*. 2006;69: 995–1000. doi:10.1021/NP050454F

19. Nielsen KF, Månsson M, Rank C, Frisvad JC, Larsen TO. Dereplication of microbial natural products by LC-DAD-TOFMS. *J Nat Prod*. 2011;74: 2338–2348. doi:10.1021/NP200254T/SUPPL_FILE/NP200254T_SI_001.ZIP

20. Hamed AA, El-Shiekh RA, Mohamed OG, Aboutabl EA, Fathy FI, Fawzy GA, et al. Cholinesterase Inhibitors from an Endophytic Fungus *Aspergillus niveus* Fv-er401: Metabolomics, Isolation and Molecular Docking. *Molecules*. 2023;28: 2559. doi:10.3390/MOLECULES28062559/S1

21. Chiang YM, Szewczyk E, Nayak T, Davidson AD, Sanchez JF, Lo HC, et al. Molecular genetic mining of the *Aspergillus* secondary metabolome: discovery of the emericellamide biosynthetic pathway. *Chem Biol.* 2008;15: 527–532. doi:10.1016/J.CHEMBIOL.2008.05.010

22. McCluskey K, Wiest A, Plamann M. The Fungal Genetics Stock Center: a repository for 50 years of fungal genetics research. J Biosci. 2010;35: 119–26.

23. Bayram Ö, Bayram ÖS, Ahmed YL, Maruyama J, Valerius O, Rizzoli SO, et al. The *Aspergillus nidulans* MAPK module AnSte11-Ste50-Ste7-Fus3 controls development and secondary metabolism. *PLoS Genet.* 2012;8: e1002816. doi:10.1371/journal.pgen.1002816

24. Fradin EF, Zhang Z, Juarez Ayala JC, Castroverde CDM, Nazar RN, Robb J, et al. Genetic dissection of *Verticillium* wilt resistance mediated by tomato Ve1*. Plant Physiol*. 2009;150: 320–32. doi:10.1104/pp.109.136762

25. Höfer AM, Harting R, Aßmann NF, Gerke J, Schmitt K, Starke J, et al. The velvet protein Vel1 controls initial plant root colonization and conidia formation for xylem distribution in *Verticillium* wilt. *PLoS Genet*. 2021;17. doi:10.1371/JOURNAL.PGEN.1009434

26. Ahmed YL, Gerke J, Park H-S, Bayram Ö, Neumann P, Ni M, et al. The Velvet family of fungal regulators contains a DNA-binding domain structurally similar to NF-κB. *PLoS Biol*. 2013;11: e1001750. doi:10.1371/journal.pbio.1001750

27. Jöhnk B, Bayram Ö, Abelmann A, Heinekamp T, Mattern DJ, Brakhage AA, et al. SCF ubiquitin ligase F-box protein Fbx15 controls nuclear co-repressor localization, stress response and virulence of the human pathogen *Aspergillus fumigatus*. *PLoS Pathog*. 2016;12: e1005899. doi:10.1371/journal.ppat.1005899

28. Gerke J, Köhler AM, Wennrich J-P, Große V, Shao L, Heinrich AK, et al. Biosynthesis of Antibacterial Iron-Chelating Tropolones in *Aspergillus nidulans* as Response to Glycopeptide-Producing Streptomycetes. *Frontiers in Fungal Biology*. 2022;2. doi:10.3389/ffunb.2021.777474

29. Leonard M, Kühn A, Harting R, Maurus I, Nagel A, Starke J, et al. *V. longisporum* elicits media-dependent secretome responses with a further capacity to distinguish between plant-related environments. *bioRxiv*. 2020; 2020.02.11.943803. doi:10.1101/2020.02.11.943803

30. Park H-S, Nam T-Y, Han K-H, Kim SC, Yu J-H. VelC Positively Controls Sexual Development in *Aspergillus nidulans. PLoS One*. 2014;9: e89883. doi:10.1371/journal.pone.0089883

31. Covert SF, Kapoor P, Lee M, Briley A, Nairn CJ. *Agrobacterium tumefaciens*-mediated transformation of *Fusarium circinatum*. Mycol Res. 2001;105: 259–264. doi:10.1017/S0953756201003872
