# Supplementary material for: The VelB IDD promotes selective heterodimer formation of velvet proteins for fungal development
Source: Life Sci Alliance. 2025 Nov 11;9(2):e202503395. doi: 10.26508/lsa.202503395 (PMC12614781; doi:10.26508/lsa.202503395)
Supplement: Supplementary file 10 [file LSA-2025-03395_TableS10.docx]

**Supplementary Tables**

**Table S10: Primers used for qRT-PCR in this study.**

| **Name** | **Gene** | **5’ – sequence – 3’** |
| --- | --- | --- |
| jg816  jg817 | *velB* A  *velB* B | CCC CTC CGT GTA TCC GTC TAA T  AGC CGA GTG CTT CAC AAG ATT T |
| kt278  kt279 | *15S rRNA* A  *15S rRNA* B | GAT CCG CGA AAA ACC TTA CCA C  TGG CAC GTC TAT AGC CCA CAG T |
| kt312  kt313 | *h2A.X* A  *h2A.X* B | TCT CGA GCT TGC TGG AAA CG  CAC CCT GGG CAA TAG TGA CG |

**References**

1. Ward JJ, McGuffin LJ, Bryson K, Buxton BF, Jones DT. The DISOPRED server for the prediction of protein disorder. *Bioinformatics*. 2004;20: 2138–2139. doi:10.1093/bioinformatics/bth195

2. Kosugi S, Hasebe M, Tomita M, Yanagawa H. Systematic identification of cell cycle-dependent yeast nucleocytoplasmic shuttling proteins by prediction of composite motifs. *Proceedings of the National Academy of Sciences*. 2009;106: 10171–10176. doi:10.1073/pnas.0900604106

3. Alvarez-Jarreta J, Amos B, Aurrecoechea C, Bah S, Barba M, Barreto A, et al. VEuPathDB: the eukaryotic pathogen, vector and host bioinformatics resource center in 2023. *Nucleic Acids Res*. 2024;52: D808–D816. doi:10.1093/NAR/GKAD1003

4. Sayers EW, Bolton EE, Brister JR, Canese K, Chan J, Comeau DC, et al. Database resources of the national center for biotechnology information. *Nucleic Acids Res*. 2022;50: D20–D26. doi:10.1093/NAR/GKAB1112

5. Harrison PW, Amode MR, Austine-Orimoloye O, Azov AG, Barba M, Barnes I, et al. Ensembl 2024. Nucleic Acids Res. 2024;52: D891–D899. doi:10.1093/nar/gkad1049

6. Sanchez JF, Entwistle R, Corcoran D, Oakley BR, Wang CCC. Identification and molecular genetic analysis of the cichorine gene cluster in *Aspergillus nidulans*. *Medchemcomm*. 2012;3: 997–1002. doi:10.1039/C2MD20055D

7. Bok JW, Chiang Y-M, Szewczyk E, Reyes-Dominguez Y, Davidson AD, Sanchez JF, et al. Chromatin-level regulation of biosynthetic gene clusters. *Nat Chem Biol*. 2009;5: 462–464. doi:10.1038/nchembio.177

8. Lo HC, Entwistle R, Guo CJ, Ahuja M, Szewczyk E, Hung JH, et al. Two separate gene clusters encode the biosynthetic pathway for the meroterpenoids austinol and dehydroaustinol in *Aspergillus nidulans*. *J Am Chem Soc.* 2012;134: 4709–4720. doi:10.1021/ja209809t

9. Nielsen ML, Nielsen JB, Rank C, Klejnstrup ML, Holm DK, Brogaard KH, et al. A genome-wide polyketide synthase deletion library uncovers novel genetic links to polyketides and meroterpenoids in *Aspergillus nidulans*. *FEMS Microbiol Lett.* 2011;321: 157–166. doi:https://doi.org/10.1111/j.1574-6968.2011.02327.x

10. Chiang Y-M, Szewczyk E, Nayak T, Davidson AD, Sanchez JF, Lo H-C, et al. Molecular Genetic Mining of the *Aspergillus* Secondary Metabolome: Discovery of the Emericellamide Biosynthetic Pathway. Chem Biol. 2008;15: 527–532. doi:https://doi.org/10.1016/j.chembiol.2008.05.010

11. Yu JH, Leonard TJ. Sterigmatocystin biosynthesis in *Aspergillus nidulans* requires a novel type I polyketide synthase. *J Bacteriol*. 1995;177: 4792–4800. doi:10.1128/JB.177.16.4792-4800.1995

12. Bouhired S, Weber M, Kempf-Sontag A, Keller NP, Hoffmeister D. Accurate prediction of the *Aspergillus nidulans* terrequinone gene cluster boundaries using the transcriptional regulator LaeA. *Fungal Genetics and Biology*. 2007;44: 1134–1145. doi:https://doi.org/10.1016/j.fgb.2006.12.010

13. Sanchez JF, Entwistle R, Hung J-H, Yaegashi J, Jain S, Chiang Y-M, et al. Genome-based deletion analysis reveals the prenyl xanthone biosynthesis pathway in *Aspergillus nidulans*. *J Am Chem Soc*. 2011;133: 4010–7. doi:10.1021/ja1096682

14. Ahmed AM, Ibrahim AM, Yahia R, Shady NH, Mahmoud BK, Abdelmohsen UR, et al. Evaluation of the anti-infective potential of the seed endophytic fungi of Corchorus olitorius through metabolomics and molecular docking approach. *BMC Microbiol*. 2023;23: 1–19. doi:10.1186/S12866-023-03092-5/FIGURES/11

15. Perlatti B, Lan N, Jiang Y, An Z, Bills G. Identification of Secondary Metabolites from *Aspergillus pachycristatus* by Untargeted UPLC-ESI-HRMS/MS and Genome Mining. Molecules. 2020;25. doi:10.3390/MOLECULES25040913

16. Liu L, Sasse C, Dirnberger B, Valerius O, Fekete-Szücs E, Harting R, et al. Secondary metabolites of hülle cells mediate protection of fungal reproductive and overwintering structures against fungivorous animals. *Elife*. 2021;10. doi:10.7554/ELIFE.68058

17. Thieme KG, Gerke J, Sasse C, Valerius O, Thieme S, Karimi R, et al. Velvet domain protein VosA represses the zinc cluster transcription factor SclB regulatory network for *Aspergillus nidulans* asexual development, oxidative stress response and secondary metabolism. *PLoS Genet*. 2018;14: e1007511. doi:10.1371/journal.pgen.1007511

18. Kralj A, Kehraus S, Krick A, Eguereva E, Kelter G, Maurer M, et al. Arugosins G and H: prenylated polyketides from the marine-derived fungus *Emericella nidulans* var. acristata. *J Nat Prod*. 2006;69: 995–1000. doi:10.1021/NP050454F

19. Nielsen KF, Månsson M, Rank C, Frisvad JC, Larsen TO. Dereplication of microbial natural products by LC-DAD-TOFMS. *J Nat Prod*. 2011;74: 2338–2348. doi:10.1021/NP200254T/SUPPL_FILE/NP200254T_SI_001.ZIP

20. Hamed AA, El-Shiekh RA, Mohamed OG, Aboutabl EA, Fathy FI, Fawzy GA, et al. Cholinesterase Inhibitors from an Endophytic Fungus *Aspergillus niveus* Fv-er401: Metabolomics, Isolation and Molecular Docking. *Molecules*. 2023;28: 2559. doi:10.3390/MOLECULES28062559/S1

21. Chiang YM, Szewczyk E, Nayak T, Davidson AD, Sanchez JF, Lo HC, et al. Molecular genetic mining of the *Aspergillus* secondary metabolome: discovery of the emericellamide biosynthetic pathway. *Chem Biol.* 2008;15: 527–532. doi:10.1016/J.CHEMBIOL.2008.05.010

22. McCluskey K, Wiest A, Plamann M. The Fungal Genetics Stock Center: a repository for 50 years of fungal genetics research. J Biosci. 2010;35: 119–26.

23. Bayram Ö, Bayram ÖS, Ahmed YL, Maruyama J, Valerius O, Rizzoli SO, et al. The *Aspergillus nidulans* MAPK module AnSte11-Ste50-Ste7-Fus3 controls development and secondary metabolism. *PLoS Genet.* 2012;8: e1002816. doi:10.1371/journal.pgen.1002816

24. Fradin EF, Zhang Z, Juarez Ayala JC, Castroverde CDM, Nazar RN, Robb J, et al. Genetic dissection of *Verticillium* wilt resistance mediated by tomato Ve1*. Plant Physiol*. 2009;150: 320–32. doi:10.1104/pp.109.136762

25. Höfer AM, Harting R, Aßmann NF, Gerke J, Schmitt K, Starke J, et al. The velvet protein Vel1 controls initial plant root colonization and conidia formation for xylem distribution in *Verticillium* wilt. *PLoS Genet*. 2021;17. doi:10.1371/JOURNAL.PGEN.1009434

26. Ahmed YL, Gerke J, Park H-S, Bayram Ö, Neumann P, Ni M, et al. The Velvet family of fungal regulators contains a DNA-binding domain structurally similar to NF-κB. *PLoS Biol*. 2013;11: e1001750. doi:10.1371/journal.pbio.1001750

27. Jöhnk B, Bayram Ö, Abelmann A, Heinekamp T, Mattern DJ, Brakhage AA, et al. SCF ubiquitin ligase F-box protein Fbx15 controls nuclear co-repressor localization, stress response and virulence of the human pathogen *Aspergillus fumigatus*. *PLoS Pathog*. 2016;12: e1005899. doi:10.1371/journal.ppat.1005899

28. Gerke J, Köhler AM, Wennrich J-P, Große V, Shao L, Heinrich AK, et al. Biosynthesis of Antibacterial Iron-Chelating Tropolones in *Aspergillus nidulans* as Response to Glycopeptide-Producing Streptomycetes. *Frontiers in Fungal Biology*. 2022;2. doi:10.3389/ffunb.2021.777474

29. Leonard M, Kühn A, Harting R, Maurus I, Nagel A, Starke J, et al. *V. longisporum* elicits media-dependent secretome responses with a further capacity to distinguish between plant-related environments. *bioRxiv*. 2020; 2020.02.11.943803. doi:10.1101/2020.02.11.943803

30. Park H-S, Nam T-Y, Han K-H, Kim SC, Yu J-H. VelC Positively Controls Sexual Development in *Aspergillus nidulans. PLoS One*. 2014;9: e89883. doi:10.1371/journal.pone.0089883

31. Covert SF, Kapoor P, Lee M, Briley A, Nairn CJ. *Agrobacterium tumefaciens*-mediated transformation of *Fusarium circinatum*. Mycol Res. 2001;105: 259–264. doi:10.1017/S0953756201003872
